# Use It and Improve It or Lose It: Interactions between Arm Function and Use in Humans Post-stroke

**DOI:** 10.1371/journal.pcbi.1002343

**Published:** 2012-02-16

**Authors:** Yukikazu Hidaka, Cheol E. Han, Steven L. Wolf, Carolee J. Winstein, Nicolas Schweighofer

**Affiliations:** 1 Computer Science, University of Southern California, Los Angeles, California, United States of America; 2 Brain and Cognitive Sciences, Seoul National University, Seoul, Republic of Korea; 3 Department of Rehabilitation Medicine, Emory University, Atlanta, Georgia, United States of America; 4 Biokinesiology, University of Southern California, Los Angeles, California, United States of America; University College London, United Kingdom

## Abstract

“Use it and improve it, or lose it” is one of the axioms of motor therapy after stroke. There is, however, little understanding of the interactions between arm function and use in humans post-stroke. Here, we explored putative non-linear interactions between upper extremity function and use by developing a first-order dynamical model of stroke recovery with longitudinal data from participants receiving constraint induced movement therapy (CIMT) in the EXCITE clinical trial. Using a Bayesian regression framework, we systematically compared this model with competitive models that included, or not, interactions between function and use. Model comparisons showed that the model with the predicted interactions between arm function and use was the best fitting model. Furthermore, by comparing the model parameters before and after CIMT intervention in participants receiving the intervention one year after randomization, we found that therapy increased the parameter that controls the effect of arm function on arm use. Increase in this parameter, which can be thought of as the confidence to use the arm for a given level of function, lead to increase in spontaneous use after therapy compared to before therapy.

## Author Summary

Although, there is now definitive evidence that intensive task-specific practice is effective for improving upper extremity function and use after stroke, it is unclear how individual patients recover from stroke, and how they respond to therapy. Here, we propose a novel computational model of stroke recovery to study the time-varying dynamics of recovery of individuals at least 3 months post-stroke with mild to moderate impairments. Our model gives support to one of the axiom of neuro-rehabilitation “use it or lose it”. Furthermore, analysis of the model parameters showed that increase in confidence to use the affected arm during therapy may affect the dynamics of recovery. Our long-term goal is to develop and validate a method based on such dynamical models, to allow clinicians and patients to make informed decisions about treatment and potentially determine the critical dose of motor therapy for an individual patient.

## Introduction

Stroke often leaves patients with predominantly unilateral motor impairments. Although the affected upper extremity is often not completely paralyzed, the recovery of upper extremity function is often achieved solely by compensatory use, i.e., choice of the less-affected arm [1]. Improving use of the more affected arm is important however, because difficulty in using this arm in daily tasks has been associated with reduced quality of life [2].

There is now definitive evidence that intensive task-specific practice is effective for improving upper extremity function and use after stroke [3], [4], [5], [6]. Such training reverses, at least partially, the loss of cortical representation due to lesion through recruitment of adjacent brain areas in animals [7], [8] and in humans [9]. This reorganization lasts several years [10], and has been linked to improved performance [11] and increased use of the affected limb [12]. On the contrary, lack of training has been associated with further loss of cortical representation [7], [13].

**Figure 1:**
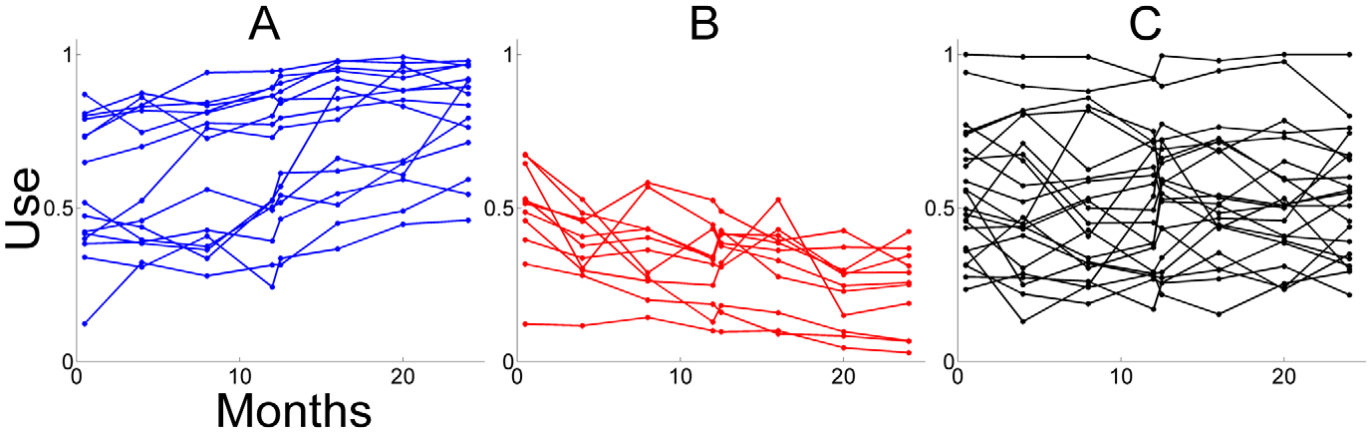
Longitudinal arm and hand use data (as measured by the MAL AOU test, normalized) for 48 participants of the immediate group in EXCITE illustrating how use can increase (A), decrease (B), or not change (C) in the 24 months following therapy. Classification in the three categories was based on the significance of the slope parameter of a linear model fit of use as a function of time, with a lenient criterion to test the hypothesis that the slope is not different from zero (p<0.25). Use increase category, N = 14; Use decrease category, N = 12; No change in use category, N = 22.

Thus, the axiom “Use it and improve it, or lose it” [14], seems appropriately applicable to the training period, when the individual is “forced” to use the affected upper extremity. But, what happens outside of therapy, when the individual is free to use, or not use, the affected limb? In some individuals, function and use further improve in the years following therapy [15], [16], [17] (see Figure 1A). For other individuals, function and use decrease in the years following therapy (see Figure 1B). We previously hypothesized that the repeated decisions to use the affected limb in daily activities may be a form of motor practice that can lead to further improvements [15]. Similarly, repeated, failed, attempts to use the affected limb have been hypothesized to underlie worsening of the impairment in a process termed “learned non-use” [18].

In our previous neuro-computational model of stroke recovery, we attempted to shed light on the interactions between function and use in general and learned non-use in particular [19]. Our model contained two independent motor cortices, each controlling the contralateral arm, with one being affected by stroke. Before each movement, one motor cortex was selected by an adaptive decision-making system, tentatively located in cortico-striatal networks. Arm performance improved via neural reorganization in the motor cortex, which learned both to minimize directional errors (via supervised learning) and to maximize neuronal activity for desired movement directions (via Hebbian learning). Furthermore, the decision to use one limb or the other was made by comparing the “action value” of each limb in the adaptive decision-making system. The values for each arm were updated based on reward prediction errors (via reinforcement learning). If performance-based rewards were greater than expected, the arm was chosen more often for this particular movement. Thus, the model predicted that function of the affected arm depends on prior use and that, in turn, arm use depends on non-linear competition between prior functions of the affected and the non-affected arm. The model also predicted that if spontaneous recovery, or motor training, or both, brings performance above a certain threshold, the repeated spontaneous arm use provides a form of motor learning that further bootstraps performance and spontaneous use. Below this threshold, spontaneous arm use after training decreases (thus the model exhibits “learned non-use”), and compensatory movements with the less affected hand are reinforced. We previously provided clinical evidence for such a threshold at the group level [20].

Here, our principal aim was to test the hypothesis that, in individuals in the chronic phase post-stroke, function of the affected arm depends on prior use of that arm and arm use, in turn, depends non-linearly on function, as predicted from our previous model. For this purpose we developed a new data-driven *quantitative* first-order dynamical model of stroke recovery that links arm function and use with a small number of parameters, which can be directly adjusted from actual data. We obtained data on upper extremity function and use for a two-year period starting from 3 months or more after stroke from the database of the Phase III randomized controlled clinical EXtremity Constraint Induced Therapy Evaluation (EXCITE) trial [3], which aimed at demonstrating the efficacy of a rehabilitative intervention for upper extremity. Arm function was derived from the time score of the Wolf Motor Function Test (WMFT) [21], [22] and arm use data was derived from the Motor Activity Log Amount of Use (MAL AOU) [23], [24]. Because of the sparsity of the data, we used Bayesian regression to fit the model. In addition, Bayesian regression allowed us to systematically compare our model with alternative models to test our hypothesis. We validated the model by computing the prediction errors of the model with a leave-one-out method.

Our secondary aim was to investigate whether motor therapy can change the hypothesized relationship between arm function and use by examining the model parameters before and after therapy. Besides improving both function and use, therapy may increase the confidence to use the arm [25], [26]. We thus predicted, that, the relationship that links arm function to arm use can be modified by therapy, and that controlling for the level of function, arm use can increases after therapy compared to before therapy.

## Methods

### Data for model parameter fit and model selection

In EXCITE, two groups of participants 3 months or more post-stroke were randomly assigned to either an immediate or a delayed Constraint Induced Movement Therapy (CIMT) group [3], [27], [28]. After 3 months, changes in function can be attributed more to learning and adaptation rather than to significant physiological modifiers that dominate the initial recovery period. The immediate group received two weeks of therapy from time Pre1 (t = 0) to Post1; the delayed group received two weeks of therapy after a one-year delay, from Pre2 (t = 1 year) to Post2.

The measure of function that we used to develop our model was the negative of the logarithm of the WMFT time score, normalized between 0 and 1. The WMFT time score [21], [22] has been used as either a primary or a secondary outcome in more than 70 published studies including the EXCITE trial. The test determines the time required for patients with stroke to perform 15 everyday tasks with each upper extremity. Tasks are sequenced so that the first six tasks involve simple limb movements, primarily of the proximal musculature; the next nine tasks require manipulation and distal control. The time score is computed by adding the times of the tasks that the subject can perform within 120 seconds. For each task that the subject cannot perform, 120 sec are added. The WMFT time score has good reliability, validity, and no learning effect [22]. Note that because the more simple tasks can normally be performed quickly, the distribution of the WMFT time score has a long-tail. The natural logarithm of the WMFT time score is therefore taken to transform the distribution into a normal distribution [3]. To readily incorporate the time score of the WMFT (after logarithm transformation) into our model, we negated the logarithm transformed WMFT score such that a good (low) WMFT time score corresponds to good (high) arm function. We then normalized the range by dividing by the difference between the highest score and the lowest score in the data set, and subtracting the lowest score in the data set from each point. Thus, a normalized score of 1 corresponds to excellent function and 0 to very poor function.

The measure of arm use that we incorporated to develop our models was the average MAL AOU score, normalized between 0 and 1. In the MAL AOU [24], [29], the participants (or their caregivers) rate how much the paretic arm is used spontaneously to accomplish 30 activities of daily living outside of the laboratory. Each item on the MAL AOU is ranked from 0 (no use) to 5 (normal) via increments of 0.5. Validity and reliability of the MAL AOU has been established [24]. The MAL AOU has been used extensively in studies with a few repeated measurements, including in the EXCITE trial.

Participants were tested with the WMFT and the MAL AOU at Pre1 (t = 0 week), Post1 (t = 2 weeks), Pre2 (t = 1 year), and Post 2 (t = 1 year+2 weeks). All participants were also tested at 4 months, 8 months, 16 months, 20 months, and 24 months. In the immediate group, because we only studied the participants' behavior after therapy, we excluded data at Pre1. Furthermore because little change in function or use is likely to happen within a 2-week-period one year after CIMT for the immediate group [16], we averaged the data at between Pre2 and Post2 for this group. Thus, for each subject of the immediate group, a total of 7 data points were available, each spaced by 4 months (at Post1, 4, 8, 12, 16, 20, and 24 months), as shown in Figure 1. In the delayed group, we compared the participants' behavior after therapy to the behavior before therapy. Because little change in function or use is likely to happen in two weeks between Pre1 and Post1 for this group [3], we averaged the function and use data at these two data points. Thus, for each subject of the delayed group, 4 data points were available before therapy (at 0, 4, 8 months, and Pre2) and 4 data points available after therapy (at Post2, and 16, 20, and 24 months).

Because of the very limited number of time points in our study, we only analyzed the data of participants with full data sets, that is, each participant had a full complement of WMFT and MAL AOU data. In the immediate group, 48 participants had a full data set. In the delayed group, 45 participants had a full dataset.

### Quantitative models of arm function and use interaction

We investigated the simplest possible model that best accounted for four essential characteristics of our previous neuro-computational model [19]: 1) Time varying changes in arm function and use reflecting the dynamic of stroke recovery. 2) Effect of use on function, with high use leading to higher future function, and low use leading to lower future function. 3) Effect of function on decision to use the arm, with higher function leading to higher future use, and lower function leading to lower future use. 4) Decision to use the affected arm or the non-affected arm based on competition between prior function of the affected and function of the non-affected arm.

We specifically hypothesized that a first order non-linear dynamical system, with two equations, can account for the interactions between arm function and spontaneous use in individuals post-stroke. The first (state-space) equation updates the function of the affected arm; the second equation updates the use of that arm.

Characteristics (1) and (2) above can be encapsulated by the evolution of arm function at time step *t* in terms of arm function and use at the previous time step as:
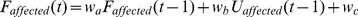
where arm function at *t*, 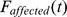, is updated based on arm function and use at the previous time step *t*−1, 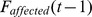 and 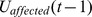, 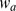 is a decay rate, 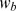 a ‘use effect’ rate, and 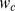 a constant input. Given the very few data points at our disposal (7 points in the immediate group), it is unlikely however that such a complex model with 3 free parameters would provide both good fit and good generalization (See sub-section “Model fit” below). Although we consider the 3-parameter model above and a simpler 2-parameter model with 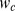 = 0 for model comparison (see below), we take as our reference model the simplest model, the 1-parameter model given by:
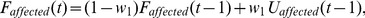
(1)where 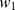 is a free parameter. Equation (1) represents a condensed version of “Use it and improve it, or lose it”, in the condition that 0≤
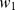
≤1: if 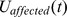 is zero or small, 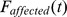 decreases. If 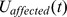 is large, then 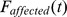 increases. The parameter 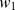 can be considered as a ‘use effect’ rate; the larger this rate, the greater the effect of spontaneous arm use on function. The term (1−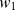) is a decay rate of arm function: with zero use, arm function would decay exponentially with time constant *D*/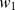, where *D* is the time step of 4 months.

Characteristics (1), (3) and (4) above can be encapsulated by the update of arm use at time step *t*, 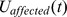, in terms of arm function in the previous time step, 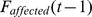 as:
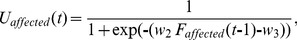
(2)where 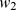 and 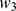 are free parameters. Equation (2) is a sigmoidal equation that arises from common decision-making models in the reinforcement-learning framework [30], in which the probability to take an action is computed by comparisons of the values of each actions, with the action with the highest “value” being the most probable. Here, we assumed that the “action value” of each limb is proportional to the function of each limb at the previous time step. The slope parameter 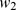 thus controls the sensitivity of arm function on arm use and can tentatively be considered as a “confidence parameter”: for equal function, greater or smaller 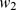 leads to more or less use, respectively. The parameter 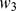 encapsulates the function of the non-affected arm 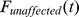 together with any non-modeled bias for preferred use of one arm versus the other, such as arm dominance or side of stroke. We did not include 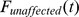 in the model because the average changes in function of the unaffected arm following therapy are relatively small compared to the average changes observed in the affected arm. Among participants of the immediate group the average log time WMFT scores is 8.62±9.78 (SD) for the affected arm, and 1.99±0.82 for the unaffected arm. The median percentage change in the score for the unaffected arm from just after therapy (Post1, t = 2 weeks) to 24 months, normalized by the score of the affected arm just after therapy, is −3.6% and the interquartile range 7.1%. By comparison, the median percentage change of the score for the affected arm from just after therapy to 24 months, normalized by the score just after therapy (Post1, t = 2 weeks) is −23.2% and the interquartile range 55.5%. We thus considered the function of the non-affected arm constant over the two years following therapy; only the function of the affected arm 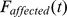 enters Equation (2) (henceforth, we drop the subscript “*affected*”).

Note that because of the simple 1-parameter model of function, arm function converges to the same value as use in the steady state (although after transformations to the original WMFT and AOU score, the values would be of course different). This is simply due to our choice of a single parameter function model, and there is no reason why this should happen in actual individuals post-stroke. Nevertheless, our model may still be adequate given 1) that the variables may not converge to their asymptotic values within two years because of long-time constants, and 2) the trade-off between fit and complexity that favors simpler models.

### Model fit, immediate and delayed groups

We estimated 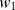, 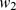, and 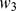 from function and use data of the EXCITE trial participants in both the immediate and the delayed group. We also aimed at testing our hypothesis of interactions between arm function and use as encapsulated in Equation (1) and (2), against a number of alternative hypotheses, as we now describe.

Because we have only 7 data points (immediate group) and 4 data points (delayed group for each before and after-therapy) for each arm function and use, we must ensure that the model does not overfit the data, that is, the model should describe the underlying relationship, not the random error or noise. Overfitting generally occurs when a model is excessively complex, such as having too many parameters relative to the number of data points. For instance, in frequentist (maximum likelihood) linear regression, a minimum of 10 or 15 points per predictor is usually considered necessary.

In contrast, Bayesian regression is the method of choice in our case, as it does not overfit the data for very small data sets (see [31] and below for rationale). The Bayesian regression framework has the additional advantage of allowing principled model comparison based on the training data alone, that is, without the need for cross-validation, which “wastes” training data. In light of these qualities, we used Bayesian regression to determine the parameters of all the candidate models based on (normalized) WMFT and MAL AOU data in the immediate group following therapy for each individual participant (*N* = 48).

Here we illustrate Bayesian regression for our reference model of Equation (1) and (2). Similar methods are used for the alternative models. We first reformulated Equation (1) and (2) to form equations linear in model parameters:
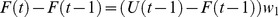
(3)

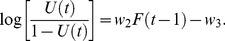
(4)We then transformed in a linear regression form:
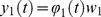
(5)

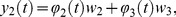
(6)where (5) and (6) correspond to (3) and (4) respectively. 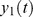 and 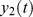 are the dependent (target) variables, representing the left-hand side of (3) and (4) respectively, and 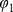, 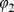, and 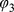 are basis functions (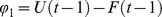, 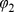 = 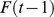, and 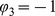). Note that we can decouple 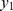 and 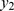 for the purpose of model parameter estimation; hence, we use 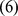 as an example in the following discussion.

Using a vector form, Equation (6) gives the regression model given model parameters:
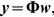
(7)where 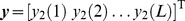, where *L* is the number of measurements, 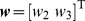, and 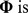 the design matrix
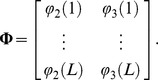
We need two consecutive measurements to estimate our regression model Therefore *L* = 7−1 = 6 for the immediate group, and *L* = 4−1 for the delayed group. Hence 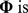 *L*×*M* matrix, where *M* is the number of model parameters (i.e., *M* = 2 for arm use model (2)).

Measurements from the EXCITE clinical data contain noise, and we assume that this noise is Gaussian added to the linear regression model ***y***. The data distribution 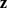 is thus assumed to be drawn independently from Gaussian distribution with mean 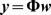 and variance 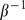:
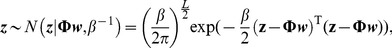
(8)where 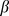 is a data accuracy hyper-parameter (inverse of variance). In Bayesian regression, we treat model parameters as a probability distribution. We assume that the *prior* distribution of model parameters is also independent identically distributed Gaussian.
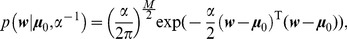
(9)where 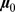 is the mean of model parameter, and 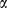 the model accuracy hyper-parameter.

The goal of Bayesian regression is to maximize the Bayesian model evidence, which is the probability of data distribution, given the model parameters: 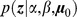. Using the sum rule and product rule of probability from (8) and (9), and taking the logarithm, we obtain the log of the model evidence (see [31] page 167 for derivation for 0 centered priors, and Supplementary material: Text S1):
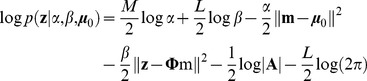
(10)

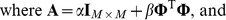
(11)

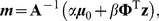
(12)where |
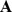
| is the determinant of 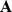, and 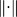 is the Euclidean norm. As shown from Equation (S24) to (S26) in Supplementary Material Text S1, 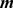 is actually the mean of the *posterior* distribution of the model parameters, and 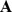 is the accuracy (inverse of covariance) of the distribution. Note how 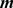 reduces to the frequentist regression solution for 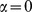.

Equation (10) illustrates how Bayesian regression implements a trade-off between data fitting and model complexity. With larger 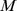 (more model parameters), 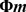 can better approximate the data distribution 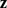, and the error between 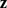 and 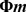, 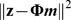 decreases. On the other hand, because the size 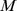 of vector 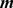 also scales up with larger number of parameters, the regularization term 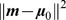 may increase. Similar trade-offs are found in (11) and (12) in the form of weighted average between prior knowledge and data. Note that, since the design matrix 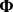 utilizes all data points, we do not need to spare testing data points for evaluating model fit, unlike cross-validation (e.g., leave-one-out).

We maximized the model evidence in terms of the two hyper-parameters 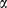, which controls model parameter distribution (9), and 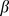, which controls data distribution (8). Note that 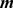 (11) and 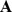 (12) are also functions of 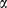 and 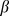. We used an iterative method [31], where we fixed 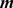 and 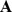 in the first step and optimized 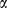 and 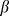, and update them with the new 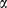 and 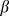 in the second step. We provide here a summary of the algorithm to compute the model evidence (see Supplementary material: Text S1 for details).

1. Set 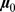, and initial 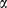 and 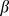

2. Compute 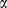 and 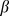 using Equation (S21) and (S23) in Text S1

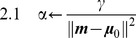


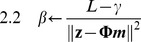
where 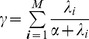 and 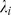 is *i*-th eigenvalue of 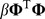

3. Update 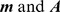 using (11) and (12)
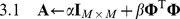


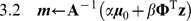

4. Repeat 2 and 3 until convergence
5. Set 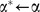 and 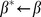, and compute model evidence (10), and posterior model parameter distribution, Equation (S24) in Text S1

### Model comparison, immediate group

Our hypothesis of “Use it and improve it, or lose it” is encapsulated in our reference model of arm function, which assumes that current arm *F(t)* function depends on a weighted sum of previous arm function *F(t−1)* and previous arm use *U*(*t*−1). We compared this model with alternative hypotheses in which *F(t)* does not depend on use, but only on previous arm function *F*(*t*−1) (i.e. use has no effect on function), or conversely, in which *F(t)* depends solely on previous use *U*(*t*−1), not on previous function. As noted above, our hypothesis is not specific to the exact model given in Equation (1), but other models containing a linear combination of *F(t−1)* and *U*(*t*−1) also fall under “Use it and improve it or lose it”. Thus, we also considered more complex linear stable models with 2 and 3 parameters. Table 1 shows the 7 possible models of function that we considered, with the bold model our “reference model”.

**Table 1:** Model comparison candidates for predicting arm function *F(t)*.

Regressors	1 parameter model	2 parameters model	3parameters model
*F(t−1)*	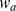 *F(t−1)*	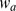 *F(t−1)+* 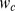	*–*
*U(t−1)*	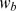 *U(t−1)*	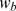 *U(t−1)+* 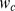	*–*
*F(t−1)* and *U(t−1)*	***(1−*** 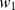 ***)F(t−1)+*** 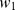 ***U(t−1)***	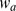 *F(t−1)+* 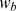 *U(t−1)*	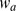 *F(t−1)+* 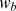 *U(t−1)+* 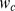 **

Our reference model of arm use assumes that current use of the affected arm *U(t)* depends via a sigmoidal function on previous function of the affected arm *F(t−1)* and a constant representing the function of the non-affected arm. We compared this model with alternative models in which *U(t)* depends linearly on previous arm function *F(t−1)*. In simulations of our previous neuro-computational model, the values for each arm were updated based on reward prediction errors at a much higher rate than the update of performance. Since our time step in the current model is 4 months, it is thus possible that the decisions to use the arm are updated much faster than performance. We therefore also compared the model of Equation (2) to models in which the current arm use *U(t)* depends on current arm function *F(t)*, either via a sigmoid or linearly. Table 2 shows the 4 possible models of use that we considered, with the bold model our “reference model”.

**Table 2:** Model comparison candidates for predicting arm use *U(t)*.

Regressors	Models
*F(t)*	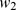 *F(t)+* 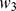
*F(t−1)*	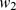 *F(t−1)+* 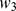
*F(t)*	*1/(1+exp[−(* 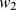 * F(t)−* 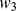 *)])*
*F(t−1)*	***1/(1+exp[−(*** 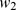 *** F(t−1)−*** 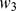 ***)])***

Initial means of the parameter distributions were taken as the values found with maximum likelihood regression of all entries of the immediate group, except the weighted average model (bold in Table 1) with initial mean value of 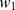 at 1. We reflected our emphasis on data and lack of prior knowledge by setting the ratio of the initial values of the prior accuracy 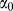 and the data accuracy 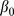 to 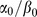 = 10^−3^ and choosing almost flat priors with 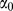 = 10^−11^, for both the function and use models. Note that these initial parameter values were taken equal for all subjects. We verified in simulations that when 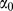
<10^−11^, the results of model comparison are qualitatively the same as that presented. We set 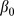 = 10^−8^ for model fitting. We also performed a sensitivity analysis (i.e. a systematic variation) on the initial data accuracy 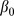 (See Supplementary Figure S1).

The Bayesian model evidence for each model was used to compare models by computing the Bayes factor (BF), which is the ratio of model evidence probability of competitive models to the reference model [32]. Thus, given the model evidence probability 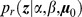 for our reference model and the model evidence probability for a competitive model 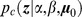, the Bayes factor is given by BF = 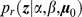/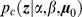. The Bayes factor has a role similar to the p-value in frequentist statistics and is used to accept or reject the hypothesis [33]. If BF<1, there is negative evidence for the hypothesis, and the hypothesis should be rejected. If 1≤BF<3, the evidence is “barely worth mentioning”. If 3≤BF<10, there is then substantial evidence for the hypothesis, and BF = 3 is a threshold for accepting the hypothesis similar to *p* = 0.05 in classical statistics. Then for BF>10, 30, and 100 there is strong, very strong, and decisive evidence for the hypothesis, respectively.

To compare the models over groups of subjects, a “group Bayes factor” can be computed by multiplying the individual Bayes factors [34]. However, such group Bayes factor is misleading in the presence of the strong outliers, which are present in our analysis due to poor convergence of the models for a number of individuals (as a result of our very limited data set). Therefore, we evaluated the number of comparisons for which BF>3 for either of the compared models to compute the “positive evidence ratio”, which serves as a measure of which model is optimal at the group level [34]. Positive evidence ratios read as *x∶y*, where *x* is the number of subject for which the Bayes factor of the reference model is greater than 3, and *y* the number of subjects for which the Bayes factor of the alternative model is greater than 3. For *N*−(*x+y*) subjects, no conclusion can be drawn.

### Model fit before and after therapy, delayed group

For this analysis, we hypothesized that motor training in the EXCITE trial, besides improving function and use, also had a “meta-learning” effect (e.g., [35], [36]). According to this hypothesis, CIMT has an effect not only on arm function and use, but also on the relationships between function and use. In our model, such meta-learning would translate to different values of the parameters 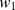, 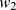, and 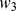 before and after therapy. In particular, we hypothesized that training increases the confidence to use the arm for a specific level of function, in which the model translates in an increase in the parameter 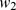. Using data from the delayed group in the EXCITE trial (*N* = 45), we used Bayesian regression for our reference model of Equation (1) and (2), and we compared the means of the parameters for each subject before and after therapy. The initial values of the hyper-parameters were the same as of the immediate group analysis.

## Results

### Model selection and fit analyses

We first computed the Bayes factors to test the two hypotheses encapsulated in Equations (1) and (2). Then we computed the positive evidence ratio for each model from the individual Bayes factors. Table 3 shows that our reference arm function model weighting previous arm function and previous use with a single parameter is strongly preferred over all other models with 2 or 3 parameters. This is presumably because of the sparsity of data in our database. Our reference arm function model is preferred over the model that depends only on previous arm function for 27 subjects out of 48 subjects, For 1 subject this alternative model is preferred, and for 20 subjects, no conclusion can be drawn. Similarly, our reference arm function model is preferred over the model that depends only on previous arm use for 25 subjects. For 5 subjects this alternative model is preferred, and for 18 subjects, no conclusion can be drawn.

**Table 3:** Positive Evidence Ratio for function model (48 subjects, immediate group).

Regressors	1 parameter model	2 parameters model	3parameters model
F(t−1)	27∶1	46∶1	–
U(t−1)	25∶5	45∶2	–
F(t−1) and U(t−1)	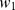 ***U(t−1)+(1−*** 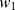 ***)F(t−1)***	47∶1	47∶1

The models correspond to the models of Table 1.

**Figure 2:**
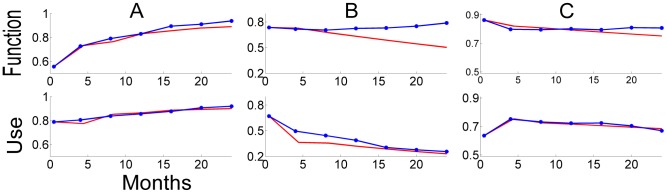
Examples of model fit for upper extremity function and use over 24 months post therapy for three subjects in the immediate group using the model of **Equation (1**) and (2) in the main text (and corresponding equations in bold fonts in **Table 1** and **2**). The blue lines show the actual data. The red lines are generated by the model with the mean model parameters, trained with 7 data points. (A) Both arm function and use improve (mean model parameters 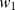 = 0.76, 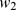 = 2.98 and 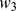 = 0.42) (B) Arm function is more or less constant, while arm use shows “non-use” (mean model parameters 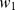 = 0.14, 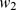 = 3.36 and 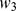 = 3.03). (C) Arm function slightly decreases, while arm use rises after 4 month and keeps the level (mean model parameters 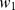 = 0.19, 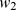 = 3.48 and 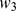 = 1.88). See how the model fit is in general good for use over the 24 months and for function in the first year but then is getting worse for function in the second year (see Table 3 and 4 for a systematic evaluation of model fit).

Table 4 shows that our reference use model with sigmoidal model of arm is strongly preferred over the two linear models. However, our reference model is not preferred over an alternative model in which arm use depends on current function; there is indeed a small advantage to the model that computes use based on current function. Figure 2 shows examples of fits with our model for both arm function and use, using the mean parameters for three subjects in the immediate group. In Figure 2A, both function and use continue to increase after therapy (mean model parameters 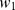 = 0.76, 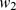 = 2.98 and 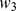 = 0.42). In Figure 2B, arm use initially largely decreases post-therapy despite relatively high function. This subject thus exhibits “learned non-use” (Mean model parameters 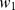 = 0.14, 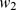 = 3.36 and 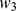 = 3.03). In Figure 2C, conversely, arm use increases after therapy, while function is relatively high. Because arm function slightly decreases in the months following therapy, so does arm use, which reaches immediately post-therapy levels after 2 years (mean model parameters 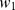 = 0.19, 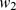 = 3.48 and 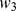 = 1.89). These figures illustrates the dynamic, nonlinear nature of arm function and use post-therapy, and how our model adequately captures these dynamical interactions and provide a reasonably good fit to the data, although the use model appears to better fit the data than the function model, and with better fit soon after therapy.

**Table 4:** Positive Evidence Ratio for use model (48 subjects, immediate group).

Regressors	Models
F(t) (linear)	38∶2
F(t−1) (linear)	38∶2
F(t) (sigmoidal)	14∶19
F(t−1) (sigmoidal)	***1/(1+exp[−(*** 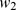 *** F(t−1)−*** 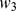 ***)])***

The models correspond to the models of Table 2.

To systematically evaluate the goodness of fit, we trained the model on 6 of the 7 data points available in the immediate group and compared the prediction of the model to the actual data point for testing (thus performing a leave-one-out model fit). Note that we kept the first and the 7th point, since we used them as an initial and final value of our model. Table 5A shows the average absolute errors of prediction among subjects of the immediate group. The average absolute errors of all 2^nd^ to 6^th^ leave-one-out prediction errors were 0.16 for arm function and 0.091 for arm use in the range between 0 and 1. The models thus reasonably fit the data, especially in the first year after therapy, although the prediction errors of the use model are lower than those of the function model overall (p<0.0001, t-test). As a comparison, the average absolute errors of randomized models were 0.22 for arm function and 0.26 for arm use (Table 5B). Here, the randomized model generates predictions points from randomly selected subject at the corresponding time step. A repeated measure ANOVA confirmed that mean prediction errors of the proposed arm function model are smaller overall than those of the randomized arm function model (p = 0.01), although the prediction errors in the proposed model increase with time (model×time interactions: p<0.0001. One way repeated ANOVAs, effect of time, proposed function model: p<0.0001, randomized function model p>0.1). Similarly, the prediction errors of the proposed arm use model are smaller overall than those of the randomized arm use model overall (p<0.001, no model×time interactions; p>0.5).

**Table 5:** Prediction errors at different time points (leave-one-out points) after therapy.

A: Prediction error of proposed model						
Leave-one-out point	2 (4 months)	3 (8 months)	4 (12 months)	5 (16 months)	6 (20 months)	Ave.
Average absolute error (Arm function)	0.097	0.12	0.18	0.18	0.19	0.16
Average absolute error (Arm use)	0.055	0.080	0.088	0.12	0.12	0.091

B: Prediction error of randomized model						
Leave-one-out point	2 (4 months)	3 (8 months)	4 (12 months)	5 (16 months)	6 (20 months)	Ave.
Average absolute error (Arm function)	0.23	0.22	0.21	0.22	0.21	0.22
Average absolute error (Arm use)	0.25	0.22	0.26	0.27	0.31	0.26

### Model parameter analysis

**Figure 3:**
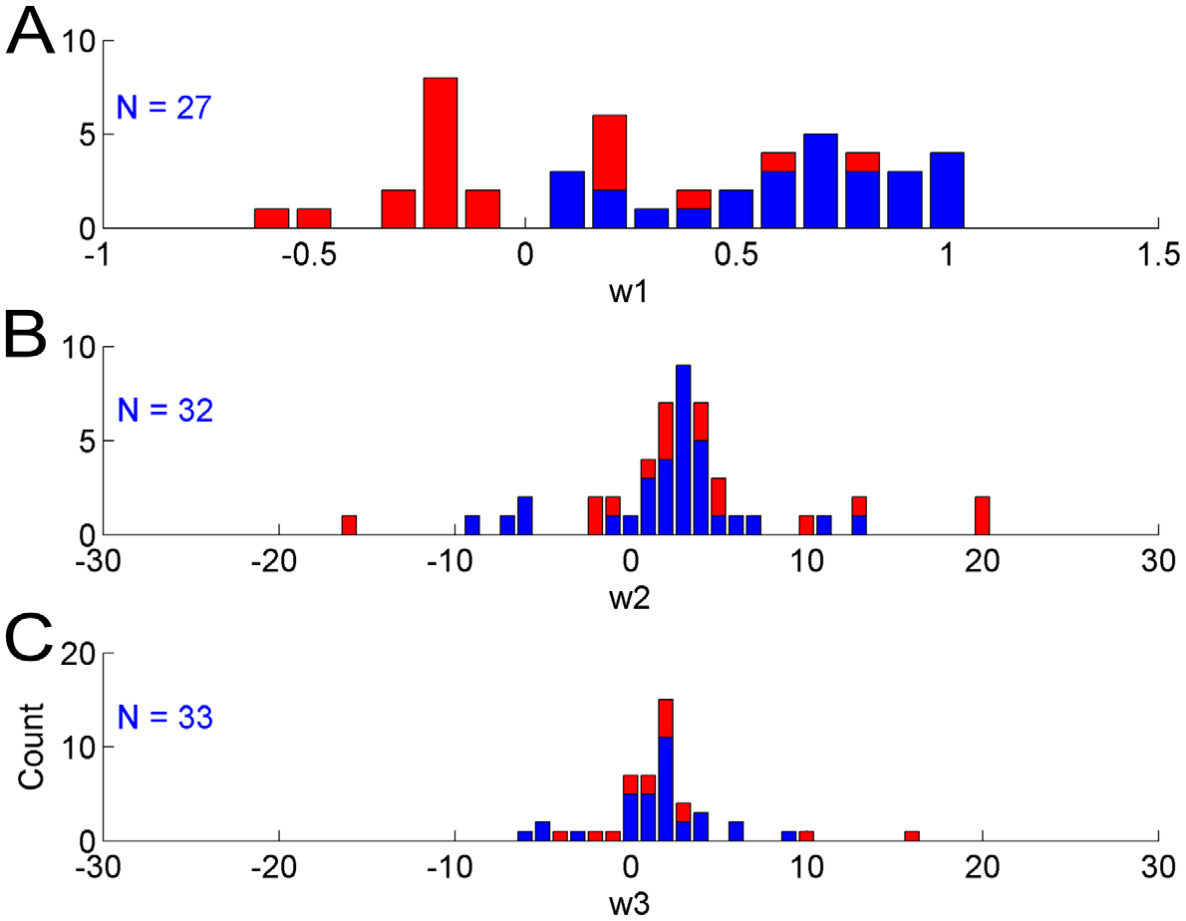
Histograms of the means of parameters 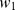
 **,** 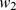 **, and** 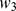 **of the model estimated with data of the immediate group in the EXCITE trial.** Blue and Red: subjects with all estimated mean parameters. Blue: subjects with mean parameters after application of convergence criteria (see Results). The numbers N's indicate the numbers of subjects with good convergence for each parameter. Note that for 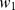, the means of all parameters with good convergence are in the range [0; 1] supporting the “Use it and improve it, or lose it” model. Similarly, for 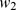, the means of most parameters with good convergence are positive, supporting an actual influence of function on use (Refer to Equation (1) and (2) in Methods for the role of these parameters in the model).

Histograms of the mean parameters 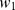, 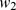, and 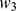 for the models of Equation (1) and (2) are shown in Figure 3. Because of the very few and noisy data points, Bayesian regression did not exhibit adequate convergence of the model parameter distributions for all subjects; that is, the parameter distributions were relatively flat for some subjects. In a first approximation, we defined good convergence as follows: the standard deviation of the final parameter distributions after convergence should be less than one standard deviation of the distributions of the parameters means. This criterion resulted in the following cut-off standard deviations: 0.316 for 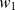; 6.38 for 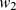 , and 3.67 for 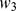. As shown in Figure 3, all negative mean parameters 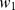 were removed after applying this criterion. Thus, for all 27 subjects with good convergence of the Bayesian regression for the function model, the mean parameter 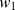 was positive and in the range [0, 1], with median 0.64. This indicates a positive effect of arm use on the previous time step upon arm function at the next time step.

Similarly, mean parameters 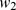 and 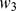 with large absolute values were removed by the cut-off procedure. The median of the mean of 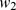 for the 32 subjects with good convergence was 2.20. The median of the mean of 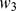 for the 33 subjects with good convergence was 1.40. Positive parameters 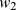 indicate that arm function has a positive effect on arm use, as hypothesized. Positive parameter 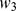 indicates competition between function of the affected limb and (constant) function of the non-affected limb, as predicted by models of decision-making based on comparisons of “values”. Note that we verified with surrogate data derived from the model that our Bayesian regression method can indeed retrieve the parameters of the original model (see Supplementary material: Text S2 and Figure S2).

### Effects of therapy on model parameters

We then examined whether CIMT had an effect on the model parameters in the delayed group by comparing before and after therapy models. Before-therapy model parameters were trained with arm function and use in the year before therapy. After-therapy model parameters were trained with arm function and use in the year after the therapy period. The standard deviation cut-off values were the same as above, and only parameters with good convergence before and after therapy were analyzed.

**Figure 4:**
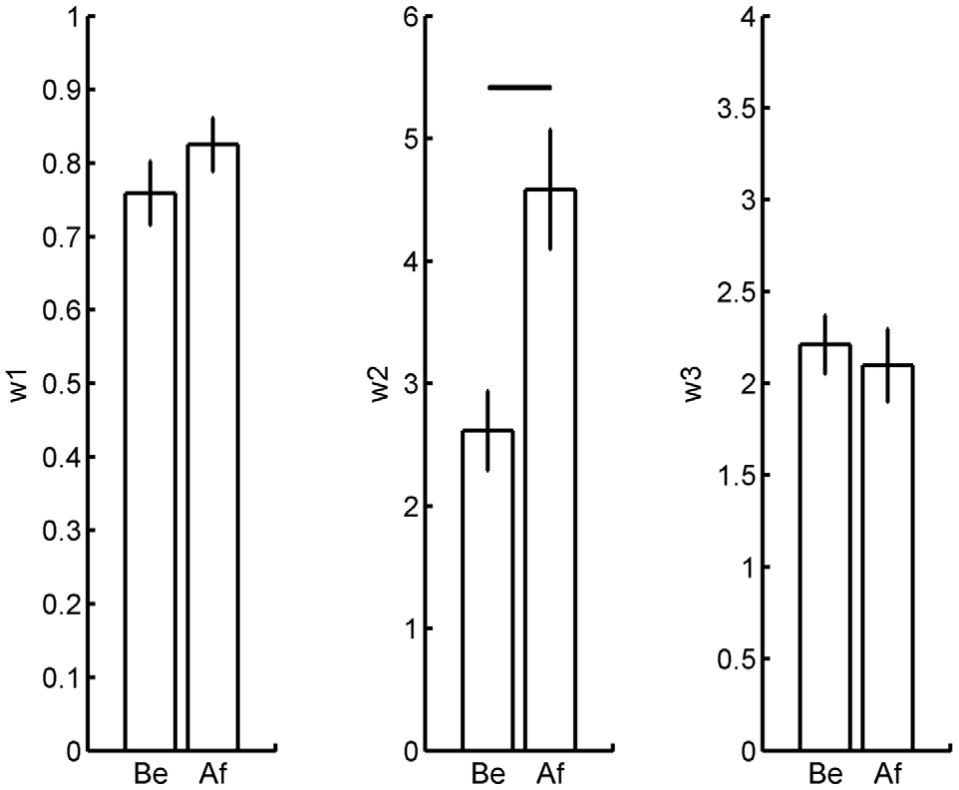
Effects of therapy on mean model parameters for participants of the delayed group in the EXCITE trial. A. Effect on 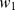. B. Effect on 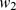. C. Effect on 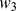. Only the mean parameter 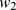 of equation 2 varies from before (Be) to after (Af) therapy. This parameter controls the effect of function on use for the affected arm. The horizontal line in B indicates p<0.05.

Among the three model parameter means, only the means of 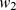 was significantly different between before and after and therapy (Figure 4B, mean of 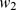 before therapy 2.95±0.32; after-therapy 4.58±0.49; p = 0.041; N = 22; 2-tailed pair t-test). There was no difference in 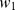 (Figure 4A, before-therapy 0.759±0.044; after-therapy 0.825±0.036; p = 0.55; N = 27; 2-tailed pair t-test) and in 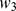 (Figure 4C, before-therapy 2.21±0.16; after-therapy 2.10±0.20; with p = 0.54, N = 28, 2-tailed pair t-test).

### Model simulations

Our previous neuro-computational model of stroke recovery [19] exhibited non-linear and bi-stable behavior of stroke recovery: the model predicted that if natural recovery, motor training or both, brings performance above a certain threshold, training can be stopped, as the repeated spontaneous arm use provides a form of motor learning that further bootstraps performance and spontaneous use.

Here, we simulated our model made of Equation (1) and (2) to study whether the simplified model of the present study also contained such threshold and bi-stable behavior, and to study the effect of the increase of the “confidence” parameter 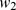 from before to after therapy, with the simplifying assumptions that therapy does not increase function and use. For this purposes we performed a parameter sensitivity analysis using the continuation and bifurcation toolbox Matcont (http://sourceforge.net/projects/matcont/).

**Figure 5:**
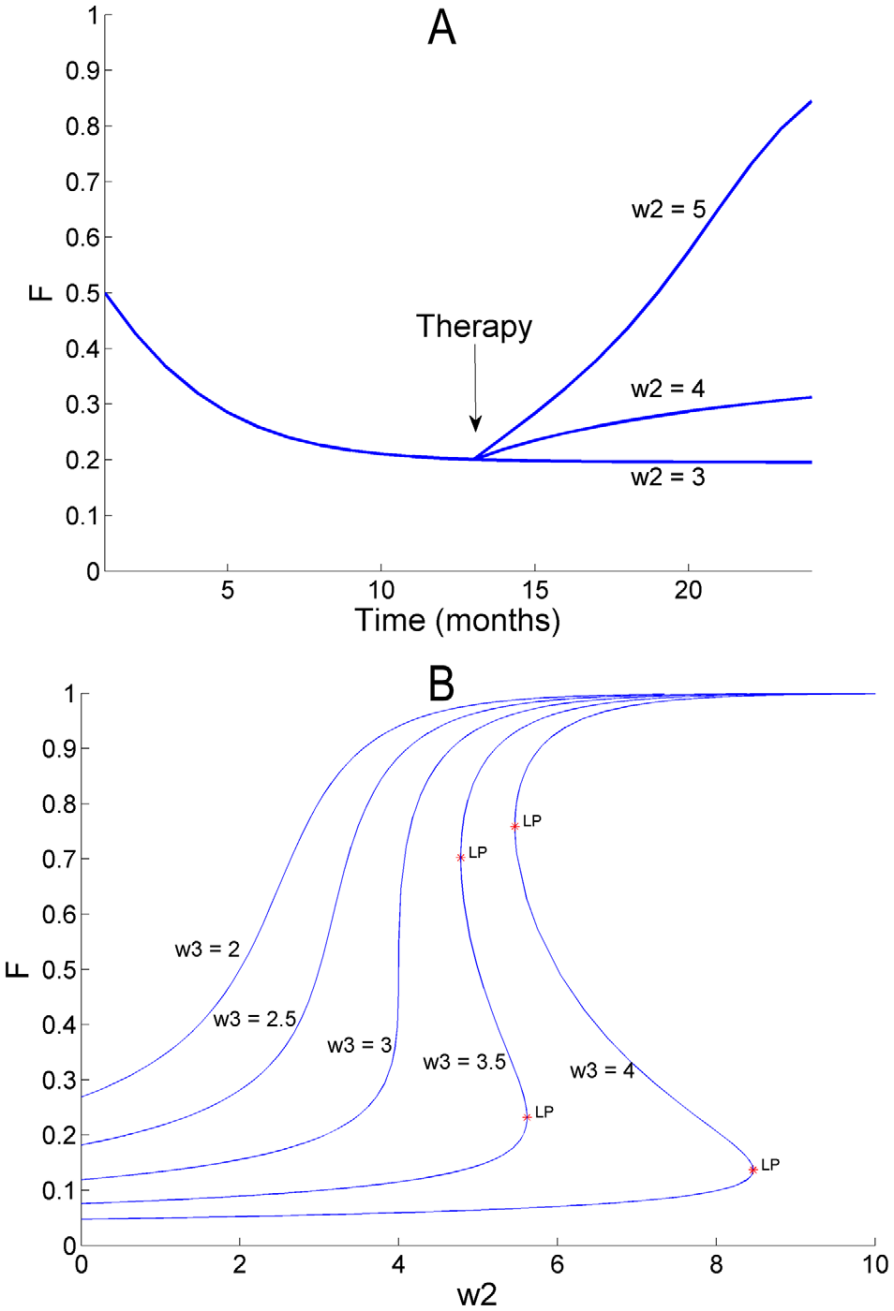
Computer simulations of arm function showing dependence on model parameters. A. Simulations of the effect on use after hypothetical changes in the confidence parameter 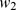 as a result of therapy. Initial parameters values: 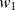 = 0.6, 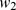 = 3, 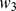 = 3. For simplicity, we assumed here that therapy has only an effect on the parameter 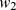 and not on use and performance (which it did in actual participants of the EXCITE trial [3]). The increase in parameter 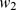 from before to after therapy parallels the increase in this parameter in the delayed group of the EXCITE trial (see Figure 4). B. Parameter sensitivity analysis showing the asymptotic value of arm function *F* as a function of parameter 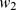 for a number of values of 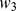. LP: limit point. The line labeled 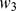 = 3 is generated by the same model as in A. For values of 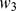
>3 the system behavior exhibits a non-stable range between the two limit points. For 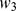 = 3.5 and 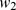 = 5 for instance, arm function F converges to either a low or a high value.

The sensitivity analysis of Figure 5B shows that for 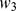
≤3 and low values of 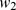, asymptotic function and use are low. However, by increasing 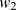, therapy can “move” the participants from one low attractor to a high attractor region, exhibiting convergence to different arm function values, as shown in simulation results of Figure 5 A and B. Thus, if therapy increases the confidence to use the arm, the greater spontaneous arm use will lead to greater function, in a virtuous cycle (Figure 5A, 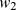 = 4 or 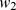 = 5). In contrast, for a low value of the parameter 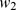, the simulated patient is in a vicious cycle and use decreases (as in Figure 5A for 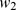 = 3). Because of competition between function or each arm in computing use, high values of 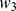 lead to greater non-use compared to smaller values of 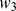 (See left side of Figure 5B). This is illustrated by comparing arm use for the two subjects in Figure 2B and 2C. The main difference in parameters between the subjects of Figure 2B and 2C is the value of 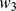. Because 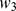 is relatively large in 2B, arm use decreases to low level; in contrast use stays relatively high in 2C. However, for 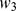
>3, a sufficient increase in the parameter 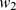 will bring the system in a truly bi-stable mode. Depending on the initial condition (i.e. values of *F(t)* and *U(t)* just after therapy), function and use can either remain near low values or near high values delimited by the low Limit Point (LP) and high LP in the Figure 5B. Thus, the model exhibits a “threshold” in function, as we proposed in our previous work [19], [20].

## Discussion

Stroke recovery is, by definition, a time-varying process. Although our dynamical “state-space” model naturally accounts for the time-varying nature of stroke recovery, this paper represents, to our knowledge, the first effort to use approach to quantitatively model recovery of individuals post-stroke. The stroke recovery model proposed here depicts a time-evolving process with interactions between arm function and use. The model, which is composed of two sub-models, one that updates arm function (Equation (1)) and the other that updates arm use (Equation (2)), has only three free parameters, which were estimated with repeated measurements of upper extremity function and use obtained in a phase III randomized controlled clinical trial, the EXCITE trial.

For a majority of the participants in the immediate group of the EXCITE trial that we studied, arm function depends both on prior function and prior use. Presumably because of the very limited amount of data that penalizes models with more parameters, the preferred arm function model performs a weighted average of previous arm function and use with a single parameter. This model is preferred for 27 subjects out of 48 over a competitive model in which arm function is not dependent on previous use, and is preferred for 25 subjects out of 48 over a model in which function is solely based on use. The alternative models are preferred for 1 and 5 subjects respectively; for the remainder of the subjects, no conclusion can be drawn. Furthermore, parameter analysis showed a positive effect of arm use at the previous time step upon arm function at the current time step, thus truly capturing the phenomenon of “Use it and improve it, or lose it” for a majority of the participants we studied. Although this phenomenon may be taken for granted by stroke rehabilitation specialists, this is, to our knowledge, the first systematic demonstration of the effect of the upper extremity use on changes in function and vice-versa in stroke recovery in individual subjects (in our previous study [20] we only study this effect of function immediately following therapy upon future use at the group level).

We further showed that for the large majority of the participants we studied, models of spontaneous arm use based on a sigmoidal dependency of arm function are preferred over linear models. This result indicates that the non-linear dependency of use on function has a strong effect on the fit of the use data. Furthermore, parameter analysis showed that arm function has a positive effect on arm use with competition between function of the affected limb and (constant) function of the non-affected limb, as predicted by models of decision-making based on comparisons of “values” e.g. [37]. Time has a lesser effect: Our reference model of Equation (2) in which use depends on previous function *F(t−1)*) is only preferred for 14 subjects over a model in which use depends on current function *F(t)*). Contrarily this alternative model is preferred for 19 subjects over the reference model (no conclusion can be drawn for the remainder 15 subjects). This inconclusive effect of time on arm use suggests that update of the arm choice (presumably via the learning of “values”) is fast compared to the update of arm function.

Our previous neuro-computational model of stroke recovery [19] exhibited bi-stable behavior of stroke recovery. Here, our simpler data-driven model also exhibits a bi-stable behavior, although for relatively large values of the parameters 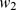 and 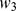 of the use model (see Figure 5B). However, even for lower value of the parameters (around the mean of the estimated parameters) therapy can, by increasing the parameter 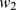, “move” the participants from one low attractor to a high attractor region shown in simulation results of Figure 5A. This simulation of the model made of Equation (1) and (2) illustrates the effect of the increase of the “confidence” parameter 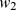 from before to after therapy, with the simplifying assumptions that therapy does not increase function and use. Simulations show that if therapy increases confidence to use the arm, the greater spontaneous arm use will lead to greater performance, in a virtuous cycle (Figure 5, 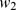 = 4 or 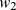 = 5). In contrast, for a low value of the parameter, the patient is in a vicious cycle and use decreases (as in Figure 5 for 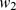 = 3). Unfortunately, because of the limited data set, the sustainability of this increase in confidence in participants of the EXCITE trial is unclear. Since the median 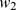 post-therapy in the immediate group (2.20) is inferior to the median 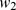 post-therapy in the delayed group (3.90) such increase may be relatively short-lasting post-therapy.

In sum, our results suggest that learned non-use results, at least in part, from three non-mutually exclusive factors: 1) a decrease in function of the affected arm; 2) a relative increase in function of the non-affected arm (if for instance stroke affects the right arm and the right-hand dominant subject is learning how to use her left arm); 3) reduced “confidence levels” in using the arm for a given function (as a result of spilling a hot coffee on someone else for instance). Since our study is only a model of changes in behavior, we can only speculate on the causes of non-use at the neural level. Reduced use may lead to contraction of motor cortical maps leading to decreased performance and further reduced use [19]; contrarily forced use (i.e. practice) may lead to map expansion and increase performance [7]. If such improvements in function together with confidence levels are sufficient, then use of the affected arm in daily activities may increase sufficiently such that function will improve spontaneously, effectively reversing non-use, as shown by our simulations in Figure 5. The median of 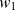 across subjects with good convergence was 0.64; given a time step of 4 months, this is equivalent to a median time constant of forgetting of 1/0.64*4 months = 6.25 months. This appears reasonable in light of the long-lasting cortical reorganization after training, e.g. [10].

Our model assumes the existence of independent measures of arm function and use across individuals at specific times. So does the MAL AOU reflect arm use that does not depend on arm function? We found a moderate but significant correlation (r = 0.58, p<0.0001) between the normalized MAL AOU vs. WMFT at t = 0 for all 93 patients (48 in immediate and 43 in delayed group). However, there is no correlation between arm function and use for those 54 patients with medium to low function (normalized WMFT<0.5, r = 0.10, p = 0.45). For this sub-group, normalized MAL AOU ranges between 0 and 0.64. This indicates that, within this sub-group, some patients have relatively high use with low function, and vice-versa, and that function and use are independent variables across subjects. Model comparisons for this sub-group of subjects with medium to low function still largely favor our hypothesized models over competitive models (See Supplementary material: Text S3).

The results of the present study need to be replicated with to-be-developed databases that contain dozens of repeated measurements of upper extremity function and use before, during, and after therapy. In particular, our model provides only “substantial” (in a Bayesian model comparison terminology) evidence for the “use and improve it or lose it” hypothesis for a majority but not for all the EXCITE participants we studied. Because of the sparcity of the data, the models did not fit the data in a satisfactory manner for large subgroups of subjects, and no conclusion can be drawn for these subjects. Furthermore, the predictions from our model, quite accurate in the first year, became worse with time across subjects (See Table 5). A possible interpretation of this result is that the influence of function on use and vice versa is stronger soon after therapy, but that this influence is reduced due to the myriad of other un-modeled factors that influence use after stroke (the patient could for instance go back to work, start to exercise, hire a caregiver, etc., all of which could affect the rate of recovery). Thus, our model is currently best viewed as a prototype against which one can develop further time dependent models of stroke recovery. Future models, based on a richer longitudinal data set of arm function and use, including measurements just after the stroke, and that include neural measurement variables such as lesion size, location, excitability of the corticospinal tract etc., might better characterize the time course of stroke recovery. Our assumptions of two independent cortices, equal roles of each arm, and pure uni-manual actions are also clear oversimplifications. Also, while motor (re-) learning after stroke can be understood at least in part as practice-dependent reduction of kinematic and dynamic performance errors [38], no such error data were available in our data set, and we therefore did not include a corresponding error-based (supervised) learning term in this simplified model (unlike in our previous model [19]). Instead, the present model only includes a trivial form of unsupervised learning in the update of arm function, and a degenerated form of reinforcement learning, with “values” simply equal to functions. Finally, our model cannot predict the time course of spontaneous recovery in the acute phase post-stroke. Here again, more longitudinal data points, including early after stroke, are needed for viable extensions of the model.

Nonetheless, our model, although preliminary and despite its important limitations, is a first step in the direction of the development of an accurate recovery model that can predict the time course of recovery post-stroke. Our long-term goal is to validate and test a method based on such dynamical models to compute the dose of arm and hand motor therapy for individual patients and provide treating therapists with such a method to be used in the clinic. A well-validated model of upper extremity recovery that generates accurate predictions of long-term use and performance, and the confidence intervals of the predictions, could be highly valuable because the clinician, patient, or provider (if applicable) will be able to make informed decisions about treatment and potentially determine the critical dose of motor therapy for an individual patient. If for instance the model predicts that no amount of recovery can increase use, rehabilitation may be in “vain”, and compensatory strategy should be emphasized. On the other hand, if therapy is predicted to be effective, a well-validated and accurate model could be used to determine minimally effective dose of therapy to maximize the benefit/cost ratio of therapy.

## Supporting Information

Figure S1Sensitivity analysis of the initial value of the data accuracy 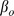 for model of arm function (A), and model of arm use (B). For this analysis, the group median of the log evidence probability among all subjects was used to represent each model performance; we then compared the model by computing a Bayes factor with the group median evidence probabilities. The x axis is the range of 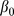 in the power of 10 and y axis is the group median of log Bayes factor for each model. Our reference model is Equation (1) for arm function, and Equation (2) for arm use; see the Table 1 in the main text for the other model entries. We varied 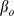 (10^−8^ 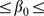
10^−3^), with fixed α = 10^−11^. The Bayes factor BF = 3 is shown by the black dashed lines in log scale. A: Sensitivity analysis of 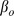 for arm function model. The bluish color lines correspond to the models of the 1^st^ row of table 1, which are regression models with *F*(*t*−1) regressor. The light blue color line shows a model with a single parameter, and the dark blue color line shows a model with two parameters. Similarly, the grayish color lines correspond to the models of the 2^nd^ row of Table 1 with regressor *U*(*t*−1). The reddish lines correspond to the models of the 3^rd^ row (with regressor *F*(*t*−1) and *U*(*t*−1)). The darker lines have the more number of model parameters. This graph shows that our reference model outperforms the others, although the differences with some models are barely worth mentioning in a small range. B: Sensitivity analysis of 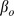 for arm use model. The bluish color lines correspond to the linear regression models. Light blue is with regressor *F(t)*, and dark blue with regressor *F*(*t*−1). The reddish color lines correspond to the sigmoidal regression models with regressor *F(t)* (light red) and with regressor *F*(*t*−1) (dark red). This figure shows that for all 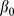
<10^−1^, the two sigmoidal arm use model largely outperform the linear models, with little differences between the sigmoidal models on one hand, and the linear models on the other hand.(DOCX)

Figure S2 Histograms of model parameter derived from surrogate data as described in Text S2. These histograms of the model parameters trained by surrogate data sets (2700 datasets for arm function and 2900 datasets for arm use) compare favorably with those derived from actual data in Figure 3. For more detail of surrogate data set, please refer to Text S2.
(DOCX)

Table S1 Positive evidence ratio of the simulation as described in Text S2. This table shows strong evidence that our proposed model performs better than the others on the surrogate data set (2700 datasets for arm function and 2900 datasets for arm use). For more detail of surrogate data set, please refer to Text S2.
(DOCX)

Table S2 A Positive evidence ratio of arm function for subjects with medium to low arm functions, as described in Text S3. This table shows model comparison results of arm function for subjects with medium to low arm function (normalized WMFT<0.5, N = 22). Model comparisons for this sub-group of subjects with medium to low function still largely favor our hypothesized models over competitive models.
(DOCX)

Text S1 Bayesian regression and model parameter optimization. Detailed derivation for Bayesian regression and optimization with respect to model parameters are provided.
(DOCX)

Text S2 Simulations with surrogate data. Numerical simulations are conducted to show that our inference procedure is able to identify which model the data came from reliably (i.e., that the model comparison works), and to recover the parameters of the true underlying model well (i.e., that the model fitting works).
(DOCX)

Text S3 Model comparison for subjects with medium and low WMFT scores.
(DOCX)
