# Supplementary figures and images for: Use It and Improve It or Lose It: Interactions between Arm Function and Use in Humans Post-stroke
Source: PLoS Comput Biol. 2012 Feb 16;8(2):e1002343. doi: 10.1371/journal.pcbi.1002343 (PMC3385844; doi:10.1371/journal.pcbi.1002343)

**Figure S1:** Sensitivity analysis of the initial value of data accuracy parameter

| 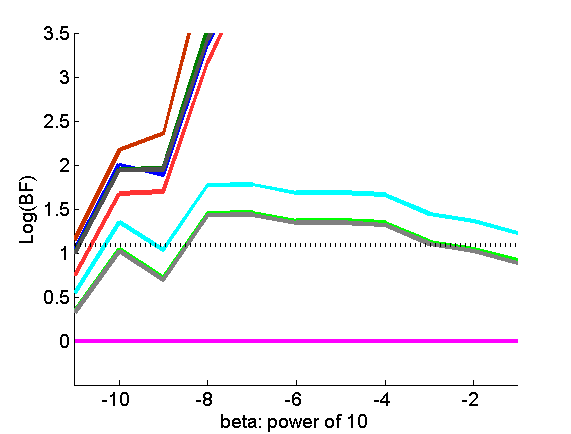 | 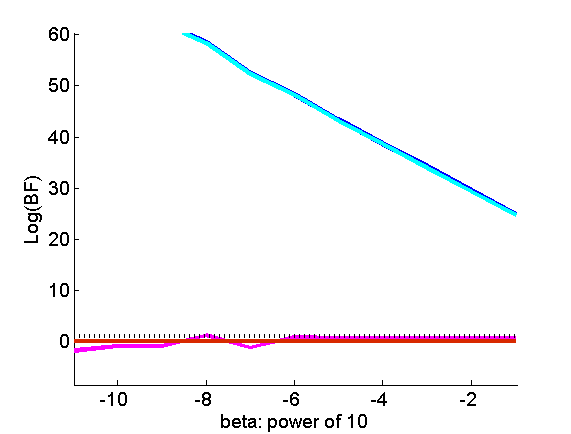 |
| --- | --- |
| A | B |

Supplement: Figure S1 — Sensitivity analysis of the initial value of the data accuracy for model of arm function (A), and model of arm use (B). For this analysis, the group median of the log evidence probability among all subjects was used to represent each model performance; we then compared the model by computing a Bayes factor with the group median evidence probabilities. The x axis is the range of in the power of 10 and y axis is the group median of log Bayes factor for each model. Our reference model is Equation (1) for arm function, and Equation (2) for arm use; see the Table 1 in the main text for the other model entries. We varied (10−8 10−3), with fixed α = 10−11. The Bayes factor BF = 3 is shown by the black dashed lines in log scale. A: Sensitivity analysis of for arm function model. The bluish color lines correspond to the models of the 1st row of table 1, which are regression models with F(t−1) regressor. The light blue color line shows a model with a single parameter, and the dark blue color line shows a model with two parameters. Similarly, the grayish color lines correspond to the models of the 2nd row of Table 1 with regressor U(t−1). The reddish lines correspond to the models of the 3rd row (with regressor F(t−1) and U(t−1)). The darker lines have the more number of model parameters. This graph shows that our reference model outperforms the others, although the differences with some models are barely worth mentioning in a small range. B: Sensitivity analysis of for arm use model. The bluish color lines correspond to the linear regression models. Light blue is with regressor F(t), and dark blue with regressor F(t−1). The reddish color lines correspond to the sigmoidal regression models with regressor F(t) (light red) and with regressor F(t−1) (dark red). This figure shows that for all <10−1, the two sigmoidal arm use model largely outperform the linear models, with little differences between the sigmoidal models on one hand, and the linear models on the other hand. (DOCX) [file pcbi.1002343.s001.docx]

**Figure S2:** Histograms of model parameter derived from surrogate data

| w1  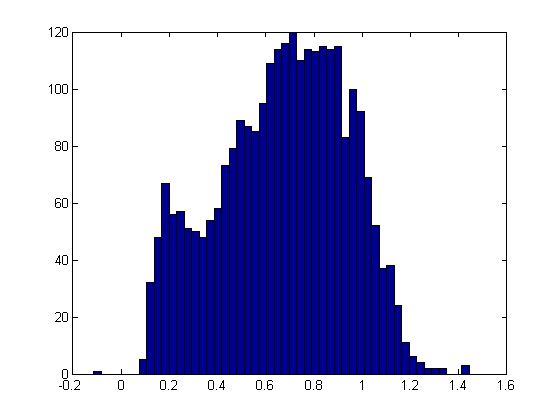 | w2  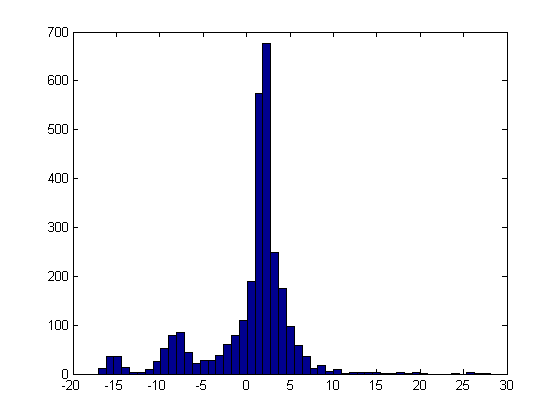 | w3  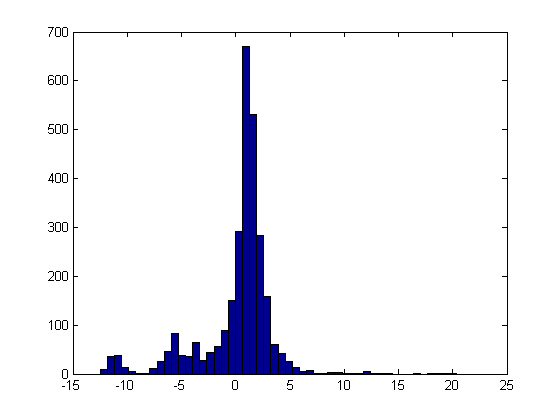 |
| --- | --- | --- |

Supplement: Figure S2 — Histograms of model parameter derived from surrogate data as described in Text S2. These histograms of the model parameters trained by surrogate data sets (2700 datasets for arm function and 2900 datasets for arm use) compare favorably with those derived from actual data in Figure 3. For more detail of surrogate data set, please refer to Text S2. (DOCX) [file pcbi.1002343.s002.docx]
