# Supplementary material for: Use It and Improve It or Lose It: Interactions between Arm Function and Use in Humans Post-stroke
Source: PLoS Comput Biol. 2012 Feb 16;8(2):e1002343. doi: 10.1371/journal.pcbi.1002343 (PMC3385844; doi:10.1371/journal.pcbi.1002343)
Supplement: Table S1 — Positive evidence ratio of the simulation as described in Text S2. This table shows strong evidence that our proposed model performs better than the others on the surrogate data set (2700 datasets for arm function and 2900 datasets for arm use). For more detail of surrogate data set, please refer to Text S2. (DOCX) [file pcbi.1002343.s003.docx]

**Table S1**

A. Positive evidence ratio of arm function models estimated from surrogate data (2700 surrogate subjects).

| Regressors | 1 parameter model | 2 parameters model | 3parameters model |
| --- | --- | --- | --- |
| F(t-1) | 1461:498 | 2228:472 | -- |
| U(t-1) | 1346:149 | 2617:81 | -- |
| F(t-1) and U(t-1) | ***(1-$w_{1}$)F(t-1)+$w_{1}$U(t-1)*** | 2549:59 | 2624:75 |

B. Positive evidence ratio of arm use estimated from surrogate data (2900 surrogate subjects).

| Regressors | Models |
| --- | --- |
| F(t) (linear) | 2689:86 |
| F(t-1) (linear) | 2424:336 |
| F(t) (sigmoidal) | 1771:486 |
| F(t-1) (sigmoidal) | ***1/(1+exp[-(****$w_{2}$* ***F(t-1)-****$w_{3}$****)])*** |
