# Supplementary material for: Use It and Improve It or Lose It: Interactions between Arm Function and Use in Humans Post-stroke
Source: PLoS Comput Biol. 2012 Feb 16;8(2):e1002343. doi: 10.1371/journal.pcbi.1002343 (PMC3385844; doi:10.1371/journal.pcbi.1002343)
Supplement: Table S2 — A Positive evidence ratio of arm function for subjects with medium to low arm functions, as described in Text S3. This table shows model comparison results of arm function for subjects with medium to low arm function (normalized WMFT<0.5, N = 22). Model comparisons for this sub-group of subjects with medium to low function still largely favor our hypothesized models over competitive models. (DOCX) [file pcbi.1002343.s004.docx]

**Table S2:**

A. Positive evidence ratio of arm function for subjects with medium to low arm function

| Regressors | 1 parameter model | 2-parameter models | 3parameter model |
| --- | --- | --- | --- |
| F(t-1) | 12:3 | 19:3 | -- |
| U(t-1) | 11:5 | 18:4 | -- |
| F(t-1) and U(t-1) | ***(1-$w_{1}$)F(t-1)+$w_{1}$U(t-1)*** | 19:3 | 19:3 |

B. Positive evidence ratio of arm use for subjects with medium to low arm function

| Regressors | Models |
| --- | --- |
| F(t) (linear) | 16:1 |
| F(t-1) (linear) | 16:1 |
| F(t) (sigmoidal) | 4:13 |
| F(t-1) (sigmoidal) | ***1/(1+exp[-(****$w_{2}$* ***F(t-1)-****$w_{3}$****)])*** |
