# Supplementary material for: Use It and Improve It or Lose It: Interactions between Arm Function and Use in Humans Post-stroke
Source: PLoS Comput Biol. 2012 Feb 16;8(2):e1002343. doi: 10.1371/journal.pcbi.1002343 (PMC3385844; doi:10.1371/journal.pcbi.1002343)
Supplement: Text S1 — Bayesian regression and model parameter optimization. Detailed derivation for Bayesian regression and optimization with respect to model parameters are provided. (DOCX) [file pcbi.1002343.s005.docx]

**Text S1: Bayesian regression and model parameter optimization**

Here, we detail the formulation of 1) the Bayesian regression for the arm function and arm use model, 2) the model evidence criterion for model fit evaluation, and 3) the iterative parameter optimization techniques to tune the model parameters.

Our state-space model in the main text Equation (1) and (2) is shown here again.

$$F\left( t \right)=\left( 1-w_{1} \right)F\left( t-1 \right)+w_{1}U\left( t-1 \right) (S1)$$

$$U\left( t \right)=\frac{1}{1+exp(-(w_{2}F(t-1)-w_{3}))} (S2)$$

where $w_{1},$ $w_{2}, \mathrm{and} w_{3}$ are model parameters to be estimated for each subject ($w_{1}$ is a “use effect” rate, $w_{2}$ a “confidence parameter” of using affected arm, and $w_{3}$ the function of the non-affected arm, as we discussed in the main text), and *t* is an time index *t=*1,…,*T* (*T*=7 for the immediate group*,* and *T*=4 for the delayed group). We omit the subscript *affected* in the *F* and *U* for simplified notation, but still *F*(*t*) is arm function of stroke-affected arm at time *t*, measured by normalized negative log WMFT, and *U*(*t*) is arm use of affected arm at time *t*, measured by normalized MAL AOU. Here, we describe how to optimize these model parameters under Bayes model evidence, given *F* and *U* measurements.

We reformulate Equations (S1) and (S2) to form equations linear in model parameters.

$$F\left(t\right)-F\left(t-1\right)=\left(U\left(t-1\right)-F\left(t-1\right)\right)w_{1} (\text{S}3)$$

$$\text{log}\left[\frac{U\left(t\right)}{1-U\left(t\right)}\right]=w_{2}F\left(t-1\right)-w_{3} (\text{S}4)$$

Then, we apply a standard linear regression form:

where (S5) and (S6) correspond to (S3) and (S4) respectively. Namely, $y_{1}\left( t \right)$ and $y_{2}\left( t \right)$ are the dependent (target) variables, representing left hand side of (S3) and (S4) respectively. The variables $\varphi_{1},$ $\varphi_{2}$, and $\varphi_{3}$are the basis functions ($\varphi_{1}=U\left( t-1 \right)-F\left( t-1 \right),$ $\varphi_{2}$=$F\left( t-1 \right),$ and$\varphi_{3}=-1$). This formulation with different basis functions and different number of model parameters is repeated to the other competitive models in Table 1 and 2. Note that we can decouple $y_{1}$ and$y_{2}$ for the purpose of model parameter estimation; hence, we use $(S6)$ as an example in the following discussion.

We now apply time-evolving measurements as a design matrix to the arm use regression model (S6) in a vector form.

$$\boldsymbol{y}=\boldsymbol{\Phi}\boldsymbol{w (}S7\boldsymbol{)}$$

where $\boldsymbol{y}=\left[ y_{2}\left( 1 \right) y_{2}\left( 2 \right) \ldots y_{2}\left( L \right) \right]^{T}$, $\boldsymbol{w=[}w_{2} w_{3}\boldsymbol{]}^{T}$, and the design matrix $\boldsymbol{\Phi}$is given by

$$\boldsymbol{\Phi}=\left[ \begin{matrix} \varphi_{2}\left( 1 \right) & \varphi_{3}\left( 1 \right) \\ \vdots& \vdots\\ \varphi_{2}\left( L \right) & \varphi_{3}\left( L \right) \end{matrix} \right]$$

Since our model is derived from 1^st^ order Markov chain, we need two consecutive measurements of U(t) and U(t+1) for the linear regression form $\left( S6 \right).$ Therefore, the number of *L* is one less than *T: L=T*-1 (*L*=6 for the immediate group, and *L=3* for the delayed group). Hence $\boldsymbol{\Phi}\mathrm{is}$ *L*×*M* matrix, where *M* is the number of model parameters (i.e., *M*=2 for arm use model (S2)).

The regression models cannot perfectly capture the dynamics, and we assume that the target variable ***y*** varies with Gaussian distribution.

$$\boldsymbol{z}=\boldsymbol{y+\eta}=\boldsymbol{\Phi}\boldsymbol{w+\eta}$$

$$\boldsymbol{z}\sim N\left( \boldsymbol{z} | \boldsymbol{\Phi}\boldsymbol{w},\beta^{-1} \right)=\left( \frac{\beta}{2\pi} \right)^{\frac{L}{2}}exp(-\frac{\beta}{2}\left( \mathbf{z}-\boldsymbol{\Phi}\boldsymbol{w} \right)^{T}(\mathbf{z}-\boldsymbol{\Phi}\boldsymbol{w})) (S8)$$

where $\boldsymbol{\eta}$ is independent identically distributed (i.i.d) Gaussian noise with the mean $\boldsymbol{\Phi}\boldsymbol{w}$ and an accuracy hyper-parameter $\left( inverse of covariance \right) \beta.$ We set the initial $\beta,$ $\beta_{0}$=10^-8^ for model fitting and vary for sensitivity analysis (see below).

In Bayesian regression, we treat model parameters as a probability distribution, not as single point values. We assume that the *prior* distribution of model parameters is also independent identically distributed Gaussian.

$$p\left( \boldsymbol{w} | \boldsymbol{\mu}_{0},\alpha^{-1} \right)=N\left( \boldsymbol{w} | \boldsymbol{\mu}_{0},\alpha^{-1} \right)=\left( \frac{\alpha}{2\pi} \right)^{\frac{M}{2}}exp(-\frac{\alpha}{2}\left( \boldsymbol{w}-\boldsymbol{\mu}_{0} \right)^{T}(\boldsymbol{w}-\boldsymbol{\mu}_{0})) (S9)$$

where $\boldsymbol{\mu}_{0}$ is the mean of model parameter, and $\alpha$ accuracy. Batch least square solutions of all subjects data from the immediate or delayed group are used for the initial mean of model parameters$\boldsymbol{\mu}_{\boldsymbol{0}}$ (except for $\mu_{0}$=1 in weighted average arm function model). Since we have little knowledge of model parameters, we set $\alpha$ very small ($\alpha_{0}={10}^{-11}$).

We reflected our emphasis on individual data and lack of prior knowledge by setting the ratio of the initial values of the prior accuracy ****and the data accuracy ****to **$\alpha_{0}/\beta_{0}$**= 10^-3^ and choosing almost flat priors with **$\alpha_{0}$**= 10^-11^. We verified in simulations that when **$\alpha_{0}$**< 10^-11^, the results of model comparison are qualitatively the same as that presented. We set **$\beta_{0}$** = 10^-8^ for model fitting and varied initial and performed a sensitivity analysis (i.e. a systematic variation) on the initial precision****.

From (S8) and (S9), we notice that the mean $\boldsymbol{\Phi}\boldsymbol{w}$ of $\boldsymbol{z}\mathrm{in}(S8)$ is not a deterministic function, since $\boldsymbol{w}$ is also a Gaussian distribution as described in (S9). This means that we cannot evaluate the model fit using point to point distance error metric; therefore, we use a Bayesian approach. We introduce a metric called Bayesian model evidence, which is the probability density function of data, given model parameters:$p\left( \boldsymbol{z} | \boldsymbol{\mu}_{0},\alpha,\beta\right)$. Using the sum rule, and product rule,

$$p\left( \boldsymbol{z} | \boldsymbol{\mu}_{0},\alpha,\beta\right)=\int p\left( \boldsymbol{z} | \boldsymbol{w},\beta\right)p\left( \boldsymbol{w} | \alpha,\boldsymbol{\mu}_{0} \right)d\boldsymbol{w} (S10)$$

Substituting (S8) and (S9) into (S10), we have

$${p\left( \boldsymbol{z} | \boldsymbol{\mu}_{0},\alpha,\beta\right)=\left( \frac{\alpha}{2\pi} \right)}^{\frac{M}{2}}\left( \frac{\beta}{2\pi} \right)^{\frac{L}{2}}\int exp(-E(\boldsymbol{w})))d\boldsymbol{w} (S11)$$

where

$$E\left( \boldsymbol{w} \right)=\frac{\beta}{2}\left( \mathbf{z}-\boldsymbol{\Phi}\boldsymbol{w} \right)^{T}(\mathbf{z}-\boldsymbol{\Phi}\boldsymbol{w})+\frac{\alpha}{2}\left( \boldsymbol{w}-\boldsymbol{\mu}_{0} \right)^{T}(\boldsymbol{w}-\boldsymbol{\mu}_{0}) (S12)$$

This is a Gaussian distribution with mean $\boldsymbol{m}_{z}=\phi\boldsymbol{\mu}_{\mathbf{0}}$and covariance $\mathbf{C}_{z}= \beta^{-1}\boldsymbol{I}_{L\times L}+\alpha^{-1}\boldsymbol{\Phi}^{T}\boldsymbol{\Phi}$. However, here, we directly evaluate integral term for derivation purpose.

We separate terms with model parameters and without them.

$$2E\left( \boldsymbol{w} \right)={\beta\left( \mathbf{z}-\boldsymbol{\Phi}\boldsymbol{w} \right)}^{T}\left( \mathbf{z}-\boldsymbol{\Phi}\boldsymbol{w} \right)+\alpha\left( \boldsymbol{w}-\boldsymbol{\mu}_{0} \right)^{T}\left( \boldsymbol{w}-\boldsymbol{\mu}_{0} \right)$$

$$=\boldsymbol{w}^{T}A\boldsymbol{w-}2\alpha\boldsymbol{\mu}_{0}^{T}\boldsymbol{w-}2\beta\mathbf{z}^{T}\boldsymbol{\Phi}\boldsymbol{w+}\beta\mathbf{z}^{T}\mathbf{z}\boldsymbol{+}\alpha{\boldsymbol{\mu}_{0}}^{T}\boldsymbol{\mu}_{0}$$

where

$$\mathbf{A}=\alpha\mathbf{I}_{M\times M}+\beta\boldsymbol{\Phi}^{T}\boldsymbol{\Phi(}S13\mathbf{)}$$

We complete the square of the model parameter terms

$$2E\left( \boldsymbol{w} \right)=(\boldsymbol{m}\mathbf{-}\boldsymbol{w})^{T}\mathbf{A}\left( \boldsymbol{m}\mathbf{-}\boldsymbol{w} \right)\boldsymbol{+}\alpha{\boldsymbol{\mu}_{0}}^{T}\boldsymbol{\mu}_{0}\boldsymbol{+}\beta\mathbf{z}^{T}\mathbf{z-}\boldsymbol{m}^{T}\mathbf{A}\boldsymbol{m}$$

where

$$\boldsymbol{m}=\mathbf{A}^{-1}\left( \alpha\boldsymbol{\mu}_{0}+\beta\boldsymbol{\Phi}^{T}\mathbf{z} \right) (S14)$$

Further, we complete the square over $\boldsymbol{\mu}$ and $\mathbf{z}$.

$E\left( \boldsymbol{w} \right)=\frac{1}{2}(\boldsymbol{w}\mathbf{-}\boldsymbol{m})^{T}\mathbf{A}\left( \boldsymbol{w}\mathbf{-}\boldsymbol{m} \right)\boldsymbol{+}\frac{\alpha}{2}\left\| \boldsymbol{m-}\boldsymbol{\mu}_{0} \right\|^{2}+\frac{\beta}{2}\left\| \boldsymbol{z-\Phi}\boldsymbol{m} \right\|^{2}$ (S15)

where $\left\| \cdot\right\|$is the Euclidean norm. We substitute (S15) into (S11), and use the fact that the integral of Gaussian distribution is equal to 1:

$$\int\frac{1}{\left( 2\pi\right)^{M/2}\boldsymbol{|A|}^{1/2}}\exp\left( -\left( \boldsymbol{w}\mathbf{-}\boldsymbol{m} \right)^{T}\mathbf{A}\left( \boldsymbol{w}\mathbf{-}\boldsymbol{m} \right) \right) d\boldsymbol{w=}1,$$

where |**A**| is the determinant of design matrix **A**. The resulting log model evidence probability is given by

$$\log p\left( \boldsymbol{z} | \boldsymbol{\mu}_{0},\alpha,\beta\right)=\frac{M}{2}\log\alpha+\frac{L}{2}\log\beta-\frac{\alpha}{2}\left\| \boldsymbol{m-}\boldsymbol{\mu}_{0} \right\|^{2}-\frac{\beta}{2}\left\| \boldsymbol{z-\Phi}\boldsymbol{m} \right\|^{2}-\frac{1}{2}log|\mathbf{A|}-\frac{L}{2}\log\left( 2\pi\right)$$

(S16)

Although (S16) looks complicated, among constant terms related with the data size $L$ and the number of model parameters$M$, we can find a trade-off between a regularization term $\left\| \boldsymbol{m-}\boldsymbol{\mu}_{0} \right\|^{2}$ and a data fitting error $\left\| \boldsymbol{z-\Phi}\boldsymbol{m} \right\|^{2}$ term, which are balanced by $\alpha$and $\beta.$ Roughly speaking, a complicated over-fitting model is penalized by the number of model dimension $M$. With larger $M$(more model parameters), $\boldsymbol{\Phi}\boldsymbol{m}$ can better approximate the data distribution$\mathbf{z}$, and the error between $\mathbf{z}$ and $\boldsymbol{\Phi}\boldsymbol{m}$, $\left\| \boldsymbol{z-\Phi}\boldsymbol{m} \right\|^{2}$ decreases. On the other hand, because the size $M$ of vector $\boldsymbol{m}$ also scales up with larger number of parameters, the regularization term $\left\| \boldsymbol{m-}\boldsymbol{\mu}_{0} \right\|^{2}$may increase. Similar trade-offs are found in (S13) and (S14) in the form of weighted average between prior knowledge and data. Note that, since the design matrix $\boldsymbol{\Phi}$ utilizes all data points, unlike cross-validation (e.g., leave-one-out), we do not need to spare testing data points for evaluating model fit.

Now, we maximize this model evidence probability in terms of $\alpha,$which controls model parameter distribution (S9), and $\beta,$which controls data distribution (S8). Note that $\boldsymbol{m}$ (S13) and $\mathbf{A}$ (S14) are also functions of $\alpha$and $\beta.$ We use an iterative method, where we fix $\boldsymbol{m}$ and $\mathbf{A}$ in the first step and optimize $\alpha$and $\beta,$ and update $\boldsymbol{m}$ and $\mathbf{A}$ with the new $\alpha$and $\beta$ in the second step (Chap. 3.5.2, Bishop, 2006). Note that this iterative method can be formulated equivalently by EM algorithm (Chap. 9.3.4, Bishop, 2006). Taking derivative of (S16) with respect to $\alpha$ is trivial in most terms but $log|\mathbf{A|}$ term. The determinant of the matrix$\mathbf{A}$ can be expressed by the product of eigenvalues of **A**: $|\mathbf{A|}$**=**$\prod_{i=1}^{M} \mathrm{eig}_{\boldsymbol{i}}\mathbf{(A)}$. Since $\mathbf{A}=\alpha\mathbf{I}+\beta\boldsymbol{\Phi}^{T}\boldsymbol{\Phi}$, the eigenvalue of **A** is given by $\mathrm{eig}_{i}\left( \mathbf{A} \right)\mathbf{=}\alpha+\lambda_{i ,}$ where $\lambda_{i}$ is *i*-th eigenvalue of $\beta\boldsymbol{\Phi}^{T}\boldsymbol{\Phi}.$ Hence,

$$\frac{d}{d\alpha}\log\left| \mathbf{A} \right|\mathbf{=}\frac{d}{d\alpha}\log\prod_{i=1}^{M} \mathrm{eig}_{i}\mathbf{(A)}\boldsymbol{=}\frac{d}{d\alpha}\sum_{i=1}^{M} \log\boldsymbol{(}\alpha+\lambda_{i}\boldsymbol{)}=\sum_{\boldsymbol{i}=1}^{M} \frac{1}{\alpha+\lambda_{i}} (S17\mathbf{)}$$

Using this, we take the derivative of (S16) with respect to $\alpha$, and set the derivative = 0.

$$\frac{d log p\left( \boldsymbol{z} | \boldsymbol{\mu}_{0,}\alpha,\beta\right)}{d\alpha}=\frac{M}{2\alpha}-\frac{1}{2}\left\| \boldsymbol{m-}\boldsymbol{\mu}_{0} \right\|^{2}-\frac{1}{2}\sum_{i=1}^{M} \frac{1}{\alpha+\lambda_{i}}=0 (S18)$$

Arranging this equation, and defining $\gamma$with ${\alpha\left\| \boldsymbol{m-}\boldsymbol{\mu}_{0} \right\|}^{2},$

$$\gamma\equiv{\alpha\left\| \boldsymbol{m-}\boldsymbol{\mu}_{0} \right\|}^{2}=M-\sum_{i=1}^{M} \frac{\alpha}{\alpha+\lambda_{i}} (S19)$$

Alternatively, we can express $\gamma$ as follows.

$$\gamma=M-\sum_{i=1}^{M} \frac{\alpha}{\alpha+\lambda_{i}}=\sum_{i=1}^{M} \frac{\alpha+\lambda_{i}}{\alpha+\lambda_{i}}-\sum_{i=1}^{M} \frac{\alpha}{\alpha+\lambda_{i}}=\sum_{i=1}^{M} \frac{\lambda_{i}}{\alpha+\lambda_{i}}\boldsymbol{(}S20\boldsymbol{)}$$

From (S19),

$$\alpha=\frac{\gamma}{\left\| \boldsymbol{m-}\boldsymbol{\mu}_{0} \right\|^{2}} (S21)$$

Similarly, taking derivative of (S16) with respect to $\beta$,

$$\frac{d log p\left( \boldsymbol{z} | \boldsymbol{\mu}_{0},\alpha,\beta\right)}{d\beta}=\frac{L}{2\beta}-\frac{1}{2}\left\| \boldsymbol{z-\Phi}\boldsymbol{m} \right\|^{2}-\frac{1}{2\beta}\sum_{i=1}^{M} \frac{\lambda_{i}}{\alpha+\lambda_{i}}$$

$$=\frac{L}{2\beta}-\frac{1}{2}\left\| \boldsymbol{z-\Phi}\boldsymbol{m} \right\|^{2}-\frac{\gamma}{2\beta}=0 (S22)$$

where we used the following equation of the derivative of $\log\left| \mathbf{A} \right|$ term with respect to $\beta.$

$$\frac{d}{d\beta}\log\left| \mathbf{A} \right| \boldsymbol{=}\frac{d}{d\beta}\sum_{i=1}^{M} \log\boldsymbol{(}\alpha+\lambda_{i}\boldsymbol{)}=\frac{1}{\beta}\sum_{i=1}^{M} \frac{\lambda_{i}}{\alpha+\lambda_{i}}$$

It is similar to (S17), but $\lambda_{i}$is a linear function of $\beta$ in $\mathrm{eig}_{i}\left( \mathbf{A} \right)\mathbf{=}\alpha+\lambda_{i}$, where $\left( \beta\boldsymbol{\Phi}^{T}\boldsymbol{\Phi} \right)\mathbf{v}_{i}\boldsymbol{=}\lambda_{i}\mathbf{v}_{i}$**,** and $\mathbf{v}_{i}$ is an eigenvector associated with $\lambda_{i}.$ Taking derivative: $\left( \boldsymbol{\Phi}^{T}\boldsymbol{\Phi} \right)\mathbf{v}_{i}d\beta\boldsymbol{=}\mathbf{v}_{i}{d\lambda}_{i}$**,** and multiplying $\frac{\beta}{\beta}$ to the left hand side, we have $\frac{1}{\beta}\left( {\beta\boldsymbol{\Phi}}^{T}\boldsymbol{\Phi} \right)\mathbf{v}_{i}d\beta\boldsymbol{=}{\frac{1}{\beta}\lambda_{i}\mathbf{v}_{i}d\beta=\mathbf{v}}_{i}{d\lambda}_{i}.$ Hence, the derivative of an eigenvalue $\lambda_{i}$with respect to $\beta$ is the ratio of them: $\frac{d\lambda_{i}}{d\beta}=\frac{\lambda_{i}}{\beta}$. Solving (S22) for $\beta$, we have

$$\beta=\frac{L-\gamma}{\left\| \boldsymbol{z-\Phi}\boldsymbol{m} \right\|^{2}} (S23)$$

 Our iterative method computes $\alpha$and $\beta$ using (S21) and (S23) in the first step, and then use the updated $\alpha$and $\beta$ to re-compute $\mathbf{A}$ and $\boldsymbol{m}$ using (S13) and (S14) in the second step. We repeat these steps until $\alpha$and $\beta$ converge. After several iterations, suppose that we have the converged values of $\alpha$and $\beta,$denoted by $\alpha^{*}$and $\beta^{*}$. This means that the model evidence log probabilityis maximized to $\log p\left( \boldsymbol{z} | {\boldsymbol{\mu}_{0},\alpha}^{*}, \beta^{*} \right)$.

Now, we can use this model evidence probability to compute the posterior probability of model parameters. Remember that the prior distribution of model parameters is given by Gaussian $\boldsymbol{w}\sim N\left( \boldsymbol{w} | \boldsymbol{\mu}_{0},\alpha^{-1} \right)$, and the data distribution is also Gaussian $\boldsymbol{z}\sim N\left( \boldsymbol{z} | \boldsymbol{\Phi}\boldsymbol{w},\beta^{-1} \right)$, in which the mean is a linear combination of $\boldsymbol{w}$. Applying a linear Gaussian formula (Roweis & Ghahramani, 1999 and Chap. 2.3.3, Bishop, 2006) of $\boldsymbol{w}$ given $\boldsymbol{z,}$ we have

$$p\left( \boldsymbol{w} | \boldsymbol{z,}\boldsymbol{\mu}_{0},\alpha^{*},\beta^{*} \right)=N\left( \boldsymbol{w} | \boldsymbol{\mu}_{\boldsymbol{w}}\boldsymbol{,}\boldsymbol{C}_{\boldsymbol{w}} \right) (S24)$$

with the mean and covariance

$$\boldsymbol{\mu}_{\boldsymbol{w}}\boldsymbol{=}\boldsymbol{C}_{\boldsymbol{w}}\left( \alpha^{*}\boldsymbol{\mu}_{0}+\beta^{*}\boldsymbol{\Phi}^{T}\mathbf{z} \right)\boldsymbol{=m} (S25)$$

$$\boldsymbol{C}_{\boldsymbol{w}}\boldsymbol{=(}\alpha^{*}\mathbf{I}_{M\times M}+\beta^{*}\boldsymbol{\Phi}^{T}\boldsymbol{\Phi}\boldsymbol{)}^{\boldsymbol{-1}}\boldsymbol{=}\boldsymbol{A}^{\boldsymbol{-1}} (S26)$$

where $\boldsymbol{m} \mathrm{and}\boldsymbol{A}$are actually given by (S13) and (S14) and $\alpha\mathrm{and} \beta$ are converged values after optimization$(\alpha\to\alpha^{*}$and $\beta\to\beta^{*})$. From this, we find that $\boldsymbol{m} \mathrm{and}\boldsymbol{A}$used in the model evidence computation above is the mean and accuracy (inverse of covariance) of the model parameter probability distribution. Note that like EM algorithm, $\alpha^{*} \mathrm{and} \beta^{*}$ is sub-optimal and hence model evidence too.

Here is a summary of the algorithm to compute Bayesian evidence with $\alpha$and $\beta$ optimization.

| 1. Set $\boldsymbol{\mu}_{0}\boldsymbol{,}$ and initial$\alpha$and $\beta$  2. Compute $\alpha$and $\beta$ using (S21) and (S23)  $2.1 \alpha\leftarrow\frac{\gamma}{\left\Vert\boldsymbol{m-}\boldsymbol{\mu}_{0} \right\Vert^{2}}$  $2.2 \beta\leftarrow\frac{L-\gamma}{\left\Vert\boldsymbol{z-\Phi}\boldsymbol{m} \right\Vert^{2}}$  where $\gamma=\sum_{\boldsymbol{i}=1}^{M} \frac{\lambda_{i}}{\alpha+\lambda_{i}}$and $\lambda_{i}$ is *i*-th eigenvalue of $\beta\boldsymbol{\Phi}^{T}\boldsymbol{\Phi}$  3. Update $\boldsymbol{m} \mathrm{and}\boldsymbol{A}$using (S13) and (S14)  3.1 $\mathbf{A}\leftarrow\alpha\mathbf{I}_{M\times M}+\beta\boldsymbol{\Phi}^{T}\boldsymbol{\Phi}$  3.2 $\boldsymbol{m}\leftarrow\mathbf{A}^{-1}\left( \alpha\boldsymbol{\mu}_{0}+\beta\boldsymbol{\Phi}^{T}\mathbf{z} \right)$  4. Repeat 2 and 3 until convergence  5. Set $\alpha^{*}\leftarrow\alpha$ and $\beta^{*}\leftarrow\beta$, and compute model evidence (S16), and posterior model parameter distribution (S24)  $5.1 log p\left( \boldsymbol{z} \vert\boldsymbol{\mu}_{0}{,\alpha}^{*},\beta^{*} \right)=\frac{M}{2}\log\alpha^{*}+\frac{L}{2}\log\beta^{*}-\frac{\alpha^{*}}{2}\left\Vert\boldsymbol{m-}\boldsymbol{\mu}_{0} \right\Vert^{2}-\frac{\beta^{*}}{2}\left\Vert\boldsymbol{z-\Phi}\boldsymbol{m} \right\Vert^{2}-\frac{1}{2}log\vert\mathbf{A\vert}-\frac{L}{2}\log\left( 2\pi\right)$  5.2 $p\left( \boldsymbol{w} \vert\boldsymbol{z,}\boldsymbol{\mu}_{0},\alpha^{-1} \right)=N\left( \boldsymbol{w} \vert\boldsymbol{m,}\boldsymbol{A}^{\boldsymbol{-1}} \right)$ |
| --- |

This discussion is based on (Chap. 3.5, Bishop, 2006 and Chap. 28, D. J. C. MacKay, 2002). For more detail, please refer to the books. It should be noted that Bayesian regression fully utilizes the data set for both model fitting and evaluation, without partitioning data for cross-validation.

We used the model evidence log probability as a metric of model fitting optimization, and applied this algorithm for individual subject data (48 subjects for immediate group, and 45 for delayed group) for all different models (9 models for arm function, and 4 models for arm use). The evidence for each model was used to compare the models by computing the Bayes factor (BF). Given the model evidence probabilities $p_{r}\left( \boldsymbol{z} | \alpha,\beta,\boldsymbol{\mu}_{0} \right)$ for our reference model and for a competitive model$p_{c}\left( \boldsymbol{z} | \alpha,\beta,\boldsymbol{\mu}_{0} \right)$, the Bayes factor is given by $p_{r}\left( \boldsymbol{z} | \alpha,\beta,\boldsymbol{\mu}_{0} \right)$/$p_{c}\left( \boldsymbol{z} | \alpha,\beta,\boldsymbol{\mu}_{0} \right)$. This Bayes factor was computed for each subject.

We evaluated Positive Evidence Ratio (PER) (Stephan, et. al., 2007), which compares the number of subjects, where the model evidence is positive (BF>=3) versus negative (BF=<1/3). Note that this criterion excludes the “barely worth mentioned” evidence (1/3< BF < 3). Since it simply compares each subject Bayes factor, this metric is robust against outliers. The result of PER was shown in Table 3 and 4 of the main text.

**References**

Bishop, C. M. (2006). Pattern Recognition and Machine Learning (Information Science and Statistics). Springer.

MacKay, D. J. C. (2002). Information Theory, Inference & Learning Algorithms. Cambridge University Press.

Roweis, S., & Ghahramani, Z. (1999). A Unifying Review of Linear Gaussian Models. Neural Computation, 345(1995), 305-345.

Stephan, K. E., Marshall, J. C., Penny, W. D., Friston, K. J., & Fink, G. R. (2007).Interhemispheric integration of visual processing during task-driven lateralization. The Journal of neuroscience, 27(13), 3512-22.
