# Supplementary material for: Use It and Improve It or Lose It: Interactions between Arm Function and Use in Humans Post-stroke
Source: PLoS Comput Biol. 2012 Feb 16;8(2):e1002343. doi: 10.1371/journal.pcbi.1002343 (PMC3385844; doi:10.1371/journal.pcbi.1002343)
Supplement: Text S2 — Simulations with surrogate data. Numerical simulations are conducted to show that our inference procedure is able to identify which model the data came from reliably (i.e., that the model comparison works), and to recover the parameters of the true underlying model well (i.e., that the model fitting works). (DOCX) [file pcbi.1002343.s006.docx]

**Text S2: Simulations with surrogate data**

The goal of these simulations was to show that our inference procedure is able to identify which model the data came from reliably (i.e., that the model comparison works), and to recover the parameters of the true underlying model well (i.e., that the model fitting works). We first generated large surrogate data sets by choosing model parameters around the means of the estimated parameters. Specifically, we selected fitted models of arm function for 27 subjects and the fitted models of arm use for 29 subjects, as these fitted models showed good convergence in parameters (see Figure 3 in main text). For each fitted model, we generated 100 surrogate data sets with parameters means drawn from normal distributions centered on the original parameter mean with standard deviation SD= 0.2.

Using these surrogate data, we performed model comparison using the same method as with the real data. As we see in Table S1 below, there is strong evidence that our proposed model performs better than the others on the surrogate data set, with qualitatively similar results to the actual data set (compare to Table 3 and 4 in the main text). Furthermore, histograms of the model parameters derived from surrogate data compare favorably with those derived from actual data in Figure 3 – see Supplementary Figure S2.
