# Supplementary material for: Use It and Improve It or Lose It: Interactions between Arm Function and Use in Humans Post-stroke
Source: PLoS Comput Biol. 2012 Feb 16;8(2):e1002343. doi: 10.1371/journal.pcbi.1002343 (PMC3385844; doi:10.1371/journal.pcbi.1002343)
Supplement: Text S3 — Model comparison for subjects with medium and low WMFT scores. (DOCX) [file pcbi.1002343.s007.docx]

**Text S3: Model comparison for subjects with medium and low WMFT scores**

We conducted model fitting on the low to medium function (normalized WMFT<0.5) subset of participants in the immediate group (N=22). Model comparison results are shown Table S2. As with the original data set, the proposed models show better performance than competitor models.
